# Transforming Cancer Research through Informatics

**DOI:** 10.1158/2159-8290.CD-24-0604

**Published:** 2024-10-04

**Authors:** Juli D. Klemm, Dinah S. Singer, Jill P. Mesirov

**Affiliations:** 1 Center for Strategic Scientific Initiatives, National Cancer Institute, NIH, Bethesda, Maryland.; 2 Department of Medicine, University of California San Diego, La Jolla, California.; 3 Moores Cancer Center, University of California San Diego, La Jolla, California.

## Abstract

For more than three decades, concurrent advances in laboratory technologies and computer science have driven the rise of cancer informatics. Today, software tools for cancer research are indispensable to the entire cancer research enterprise.

## Introduction

Improving our understanding and treatment of cancer requires unraveling its complexity at many scales. The wide-ranging clinical manifestations; the diverse environmental triggers; the multitude of genetic, genomic, and epigenetic drivers; and the heterogeneity of individual tumors have all driven the need to study cancer with ever-increasing depth, breadth, and precision. Corresponding progress in new, sophisticated high-throughput data acquisition technologies has driven advances in the informatics methods and software necessary to process, manage, analyze, and integrate these data and derive biological insights. Together, these factors have dramatically accelerated the development of software for cancer research. Software implementing informatics methods is now essential to all areas and aspects of cancer research. It is used to control instruments, track experimental parameters and protocols, manage and process raw data, perform data analysis, enable exploratory visualization, and more. These applications cut across the cancer continuum, with sophisticated tools supporting clinical trials, biobanking, cancer imaging, and high-throughput “omics” technologies. Across all these domains, from cancer discovery research to clinical research, to translation to the clinic, computational technologies have spurred the emergence of novel hypotheses and have led to profound new insights into cancer initiation, progression, and metastasis. This commentary steps through the technological evolution that resulted in the essentiality of informatics in cancer research, discusses the coevolution of data generation and data analysis technologies, and offers a vision for the future.

## The Origins of Cancer Informatics

The rising importance of software for cancer research has its origins in several transformative events over the past several decades with the convergence of computer science, technology development, and biomedicine. Whereas computational biology has its early origins in protein analysis, the field of bioinformatics emerged in earnest in the early 1990s as an increasing volume of DNA sequence data became available through resources such as GenBank. In parallel, a revolution in computer science was occurring with the creation of the World Wide Web, the exponential decrease in data storage costs, and the broad availability of personal computers. Prior to these developments, GenBank was distributed to the research community in print and then on CD-ROM. It was in this context that the NCBI website was initiated in 1994, providing online access to biomedical databases along with the alignment tool BLAST, leading it to become the most cited bioinformatics tool in history (1). In 1991, the start of the Human Genome Project, and the concomitant development of high-throughput sequencing methods, drove an accelerated need for specialized software tools to process and manage data being generated by this ambitious program. Collection, analysis, annotation, and storage of the ever-increasing amounts of mapping and sequencing data in publicly accessible, user-friendly databases were critical to the project’s success. In addition, the community needed methods and software that would allow them to extract, view, annotate, analyze, and interpret genomic information efficiently.

Technology continued to drive the field of bioinformatics when the development of microarray technology in the mid-1990s made it possible to acquire and analyze the expression, and later the structure, of thousands of genes in a single experiment. The ability to measure global, tumor-specific disease signatures had an immediate impact on cancer research. Two seminal articles describing the early application of this powerful new technology involved cancer studies, including screening for *BRCA1* mutations and analyzing expression changes in human melanoma cell lines (2, 3). As this technology evolved, gene expression profiling with microarrays fundamentally shifted the paradigm for defining tumor subtypes, as exemplified by the elucidation of breast tumor subtypes (4) and the expression-based classification of acute myeloid leukemia and acute lymphoblastic leukemia (5). The availability of this level of data generation to a single research laboratory spurred the need for new approaches to data processing, analysis, and visualization. The size and complexity of microarray data, together with important considerations for identifying and estimating errors and sources of variation, required new computational approaches and associated software. These demands drove the development of widely used informatics resources and analysis platforms still in use today, including Bioconductor (bioconductor.org), GenePattern (genepattern.org), Gene Set Enrichment Analysis with its Molecular Signatures Database (gsea-msigdb.org), Galaxy (usegalaxy.org), and WebMeV (webmev.tm4.org), among others, as well as novel visualization approaches such as clustered heat maps (6).

The accelerating efficiency and decreasing costs of sequencing, together with advances in high-throughput molecular characterization and information science, enabled the NCI and National Human Genome Research Institute to jointly initiate The Cancer Genome Atlas (TCGA) program in 2006. The goal of this massive project was to comprehensively characterize multiple molecular aspects of 33 selected cancer types, including DNA sequences (exomes for all and whole genomes for a subset), copy number and methylation, mRNA and microRNA (miRNA) expression, and the abundance of select proteins. The availability of this unprecedented dataset, in conjunction with the advancement of tools to analyze, integrate, and view these data, has had a remarkable impact on cancer research and transformed our understanding of the molecular basis of cancer, revolutionized how cancer is classified, and led to novel clinical trials. TCGA catalyzed considerable growth and advancement in computational biology by supporting the development of high-throughput genomic characterization technologies, generating a massive quantity of data, and building teams of researchers to analyze the data. Resources such as cBioPortal (cbioportal.org), the Integrative Genomics Viewer (igv.org), and the Cancer Imaging Archive (cancerimagingarchive.net) are just a few examples of software driven by TCGA that remain in wide use today. Data generated through other flagship, large-scale cancer molecular characterization initiatives including the Therapeutically Applicable Research to Generate Effective Treatments Program (pediatric data), Clinical Proteomic Tumor Analysis Consortium, and the International Cancer Genome Consortium also spurred novel analysis tools and methods.

Important data resources generated along with the rich molecular studies in TCGA were the collections of clinical diagnostic images, both pathology and radiology, available for many of the patients represented in TCGA. TCGA imaging datasets contain hematoxylin and eosin–stained images from more than 9,000 patients and radiology images from more than 1,800 patients. This resource has led to numerous studies in terms of both novel methodology development and scientific inquiry. By integrating these imaging data with the other data types collected by TCGA, a research community dedicated to linking cancer phenotypes to genotypes emerged, resulting in significant advancements and insights in the field. Although it is important to recognize that TCGA data have limitations and do not represent the diversity of the population, their availability has been catalytic for the field of cancer informatics.

## Dedicated Support for Scientific Software

Motivated by large data-generating initiatives and new measurement technologies, the 1990s and early 2000s saw a significant rise in the development of tools to manage and analyze biomedical data. Acknowledging the increasing importance of these tools and their impact on the research enterprise, a report from an NIH Working Group on Biomedical Computing (Advisory Committee to the NIH Director) published in June 1999 found, “Where software has shown itself to be valuable to a range of researchers in biomedical computing, the NIH needs to find ways to support its full development….It is time for the NIH to recognize the importance of both the tools and those who build them.” Following this report, in 2002, the NIH published the first NIH funding opportunity (PA-02-141), specifically focused on supporting scientific software. This funding opportunity, which remained active until 2014, supported the development of 143 software packages, many of which are still active and in use today.

With the rapid expansion of high-throughput data generation from samples of patients with cancer and questions specific to the complex multidimensional nature of tumor biology, it became apparent that informatics methods and software specific to cancer research were essential. In 2012, recognition of this sparked an Institute of Medicine–organized workshop on “Informatics Needs and Challenges in Cancer Research” to examine the current challenges and to make recommendations for this domain (7). In a similar timeframe, the NIH Advisory Council to the Director formed a Data and Informatics Working Group to examine these issues across the NIH institutes. Some common recommendations emerged from these discussions, including enhanced support for community-driven software, a strategy to support software throughout the development lifecycle, and enhanced peer review with sufficient representation of the user community. It was against the backdrop of these reports that the NCI proposed the development of an Informatics Technology for Cancer Research (ITCR) program, which was launched in 2013. The driving vision for the program was to advance informatics technology to enable the acquisition, integration, and analysis of cancer research data and increase knowledge across all cancer research domains to accelerate our understanding of cancer and to improve patient treatment and clinical care delivery. Since the ITCR program was established, more than 160 projects have been supported that generated some of the most widely used tools in cancer research. Whereas NCI funds the development of informatics methods and tools for cancer research through a variety of additional programs and mechanisms across the continuum of cancer research, ITCR is unique in its support for successive stages of software development (early development, hardening, enhancement, and sustainment) with an emphasis on dissemination and collaboration.

## The Rising Impact of Software for Cancer Research

In today’s data-driven age of cancer research, computational biology is integral to advancing our understanding and treatment of cancer by enabling the analysis of large, increasingly complex, and heterogeneous data. It is difficult to identify an area of cancer research in which novel computational methods have not been essential. The development of powerful new laboratory technologies has seen a corresponding explosion in the development of analytic methods. For instance, the emergence of bioinformatics tools for the analysis of single-cell sequencing data has improved our understanding of intratumoral heterogeneity, metastasis, and therapeutic resistance. More recently, single-cell technologies, together with spatial multiplex *in situ* methods, are providing the opportunity to interrogate tumor complexity at even higher resolution, advancing our understanding of the tumor microenvironment, cell–cell interactions, and other spatial features that drive cancer initiation and progression (8). These technologies are also enabling the development of tumor atlases, through efforts such as the Human Tumor Atlas Network, requiring new computational approaches to map between cellular and spatial profiles, integrate with clinical data, and find recurrent, higher order features by using computational and statistical means to identify common attributes across tumors (9). In the realm of medical imaging, computer vision methods are revolutionizing image analysis, and the last decade has seen increased development of informatics tools that convert images into quantitative data (radiomics), and their subsequent analyses with machine learning (ML) methods, informing early cancer detection and improved diagnosis, as well as treatment response and outcome prediction (10). Likewise, the digitizing of whole-slide images of tissue has led to the advent of ML tools that enable the identification and mining of subvisual features in the context of pathology (11). Additional research opportunities have also been advanced by the increased use of electronic medical records that contain real-world data on patient treatment, testing, and outcomes. Recent revolutionary developments in natural language processing, including large language models, are enabling extraction and longitudinal analysis of this information with extraordinary precision, supporting large-scale studies of tumor and cancer characteristics, clinical trial matching, and pharmacoepidemiology (12). Continued development of new informatics approaches and maintenance of foundational software and databases has become central to conducting modern cancer research.

## Supporting and Sustaining Open-Source Software for Cancer Research

Although much progress has been made in supporting software development and maintenance for cancer research, critical challenges remain. Considering the importance of informatics tools to cancer research, it is essential that the individuals and teams developing these tools receive appropriate credit and recognition for professional advancement and continued support. Many developers follow the academic model of publishing a paper to describe a new software tool, but the subsequent activities required to maintain the software often go unrecognized. Although users are encouraged by tool developers to cite the original article describing the software, such citations are often not made. These difficulties with recognition, together with competing, lucrative jobs in the industry, can lead to challenges in maintaining a workforce skilled in software engineering within the cancer research community. Recognizing the need to support the career paths of these and other critical staff, the NCI launched the R50 Research Specialist Award in 2016. These grants support experienced scientists who are not independent investigators, who often include senior software engineers working in academic research laboratories. Fostering community among these professionals is another important component to maintaining this workforce. The Research Software Alliance was convened in 2019 to advocate for the recognition of research software as a fundamental and vital component of research worldwide.

A key challenge is sustaining support for scientific open-source software tools. The research settings in which these tools are developed provide an environment where development is closely aligned with the driving research needs that are fundamental to software impact. Indeed, open-source community development may be used to assess accuracy and performance of methods. Making software readily available allows for broad testing and cross-comparison of methods in meta-analyses and benchmark datasets generated in the research community. However, the ongoing maintenance and support requirements for a widely used tool can lead to both technical and financial challenges as noted above. The organic evolution of these tools, wherein new functionality may come at the expense of technical stability, can lead to increasing challenges for its user base. Moreover, keeping up with evolving technology (hardware platforms, languages and compilers, operating systems, development tools, etc.) can be burdensome. In some cases, the individual or team that initiated the project may not have the skills or desire to address these mounting technical considerations, and the software may become unusable. Licensing to commercial concerns is one approach to sustaining scientific software and is appropriate for some tools, but for others, there is the risk that the software will diverge from the needs of the cancer research community. From a financial perspective, funders are faced with decisions about the balance of investing in sustainment of existing, widely used tools versus investing in novel informatics tools and methods that, although untested, could advance new opportunities in cancer research. Identifying appropriate approaches to addressing these sustainment considerations is an increasing priority for both tool developers and funders and is an active topic of discussion across this community.

## Looking Ahead

The rate of progress in cancer informatics today is extraordinary. Analogous to the convergence of technical advances in the 1990s that drove the beginning of the bioinformatics era, we are in the midst of a sea change in biomedical informatics driven by the massive increase in multimodal, multiscale cancer data together with groundbreaking advances in AI methods and the availability of increased computing power through Graphics Processing Units (GPUs) and exascale computing. These simultaneous events are revolutionizing all aspects and scales of biomedical research. Whereas ML methods have been used in cancer research for decades, recent developments, such as attention-based deep learning models and generative AI methods, are transforming approaches for elucidating protein structures, identifying imaging biomarkers, and building predictive models that combine molecular, spatial, and clinical data (13). Foundation models, trained on enormous corpuses of biomedical data, are emerging as powerful resources for research that can be refined with more specific datasets through transfer learning to advance research in data-limited domains, such as rare tumors (14).

At the same time, advances in measurement technologies for elucidating the underlying mechanisms of cancer continue to drive methodological advances in cancer informatics. For instance, technology for spatial interrogation of tumor tissues now supports joint analysis of histology, gene structure, gene expression, protein expression, and metabolites at nearly single-cell resolution. Coanalysis of these data is driving novel insights into cancer progression by elucidating the complex interplay among the components of the tumor microenvironment. Insights derived from these spatial studies are having direct clinical impact, informing new paradigms for cancer treatment regimens (15). These unprecedented, high-dimensional datasets have led to an explosion of novel paradigms and software tools for the data fusion and data visualization requirements to support the research questions that these data enable.

In parallel with these transformative technical advances is a heightening awareness of the ethical and equity considerations surrounding the use of AI/ML and other advanced informatics methods. These considerations are multifaceted and include concerns about the lack of representative datasets for developing these new models and methods; the risk of amplifying biases in existing datasets; the lack of transparency and explainability of many AI models; and the unequal availability of resources and training to apply these technologies. Efforts to harness these technologies to improve cancer outcomes must be balanced with efforts to mitigate the potential risks and the need to empower diverse communities to participate in the innovation ecosystem. Building a cancer research workforce that can leverage these new methods is also critical to ensuring we can realize the promise of these technologies to improve cancer outcomes.

Looking to the horizon, we can anticipate even more data science and technological advances that will accelerate data-driven cancer research. The new NIH Data Sharing and Management Policy, which now requires that all data derived from NIH funding be shared, will further increase the availability of research data for integration and analysis. In the computing realm, quantum computing is still in its infancy, but if the promise is realized, this technology would cause a fundamental shift in how we approach computation through its ability to perform calculations that are not feasible with today’s processors. Further coevolution of assay technologies, data availability, analytical methods, and computational paradigms will enable such revolutionary approaches as “digital twins”—predictive *in silico* models that are dynamically updated with physical information that offer the potential to improve medical decision-making at the patient level. The technological advances of the past three decades have deepened our knowledge of the complexity of cancer. By continuing to make progress in data-driven research, we can work towards ending cancer as we know it.
